# G4-DNA formation and chromatin remodelling are interdependent in human cells†

**DOI:** 10.1039/d3sc02533k

**Published:** 2023-06-27

**Authors:** Nicholas B. Lawler, Arnold Ou, Jessica J. King, Cameron W. Evans, K. Swaminathan Iyer, Nicole M. Smith

**Affiliations:** a School of Molecular Sciences, The University of Western Australia Perth WA Australia cameron.evans@uwa.edu.au swaminatha.iyer@uwa.edu.au nicole.smith@uwa.edu.au; b School of Physics, Mathematics and Computing, The University of Western Australia Perth WA Australia

## Abstract

DNA G-quadruplexes (G4s) have been identified as important biological targets for transcriptional, translational, and epigenetic regulation. The stabilisation of G4s with small molecule ligands has emerged as a technique to regulate gene expression and as a potential therapeutic approach for human diseases. Here, we demonstrate that ligand stabilisation of G4s causes altered chromatin accessibility dependent on the targeting specificity of the molecule. In particular, stabilisation of a target G4 using the highly specific GTC365 ligand resulted in differential accessibility of 61 genomic regions, while the broad-targeting G4 ligand, GQC-05, stabilised many G4s and induced a global shift towards increased accessibility of gene promoter regions. Interestingly, while we observed distinct effects of each ligand on RNA expression levels and the induction of DNA double-stranded breaks, both ligands modified DNA damage response pathways. Our work represents the dual possibility of G4-stabilising ligands for specific or global chromatin modulation *via* unique targeting characteristics.

## Abstract 2

DNA G-quadruplex-stabilising ligands can induce global or specific changes in chromatin accessibility and the transcriptome depending on the targeting specificity of the molecule.

## Graphical abstract

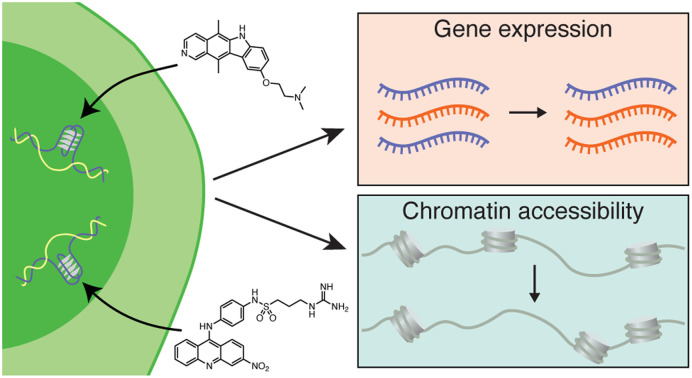

## Introduction

Nuclear processes such as gene expression are influenced by a range of mechanisms, including the arrangement of nuclear architecture. The reorganisation of nuclear architecture *via* dynamic transitions between decondensed transcriptionally active euchromatin and condensed heterochromatin is fundamental in regulating interactions of transcription factors with DNA. The principal factors governing this reorganisation in turn cause gene activation or silencing by regulating accessibility of gene promoters. Epigenetic modifications, mutations, and DNA secondary structures have all been investigated for their role in influencing chromatin accessibility and gene transcription.^1–3^ Of the various secondary structures that have been associated with chromatin accessibility and nuclear processes, G-quadruplexes (G4s) are particularly interesting as they have been identified as therapeutic targets for diseases including cancer^4,5^ and neurodegenerative disorders.^6^ G4s are sequence-specific structures that consist of stacked G-tetrads, each composed of four Hoogsteen hydrogen-bonded guanines in a planar arrangement.^7^ Unlike epigenetic and point mutations in the genome, which are heritable and stable modifications,^8^ G4 formation is dynamic and transitory. The propensity for DNA G4 formation in the human genome is very high, with over 10 000 G4-forming regions in chromatin and over 700 000 G4-forming regions identified in naked genomic DNA.^9,10^

G4 structures are enriched in gene promoters and nuclease hypersensitive regions, coincide with binding sites for transcription factors and DNA methyltransferases, and are implicated in transcriptional regulation, replication, and genomic instability.^5,10–18^ Targeted interaction and stabilisation of specific G4s using small molecules has been widely accepted as a therapeutic strategy.^15,16,19–22^

Stabilisation of G4s using small molecules has been identified as an important approach to regulate gene expression, but G4 stabilisation can increase DNA damage in cells if G4s are not efficiently regulated.^23,24^ Chromatin accessibility is required for transcription, and remodelling of chromatin is central to the DNA damage response to facilitate access of repair factors for removal of the lesion.^25–28^ Recently, G4 formation has been correlated with DNA damage repair and gene activation.^18,29–31^ Thus, G4s may contribute to the modulation of chromatin organisation, as reported by Zyner *et al.*^32^ In this work, we show that small molecule-induced G4 stabilisation can be used as a strategy to regulate chromatin remodelling in human cells. In particular, we show that G4 stabilisation and chromatin remodelling are interdependent phenomena. Enhanced chromatin remodelling of specific gene promoter regions was observed for the case of targeted G4 stabilisation using a ligand specific for the G4 in the promoter region of human telomerase reverse transcriptase (*hTERT*). On the other hand, broad-targeted, genome-wide stabilisation of G4s causes a global shift towards increased promoter accessibility, although few regions are consistently altered. Our findings using Assay for Transposase-Accessible Chromatin sequencing (ATAC-seq) and RNA-sequencing (RNA-seq) analysis coupled with G4 formation predictions in MCF-7 human breast adenocarcinoma cells reveal that G4s are key regulators of chromatin remodelling in human cells.

## Results and discussion

**Fig. 1:**
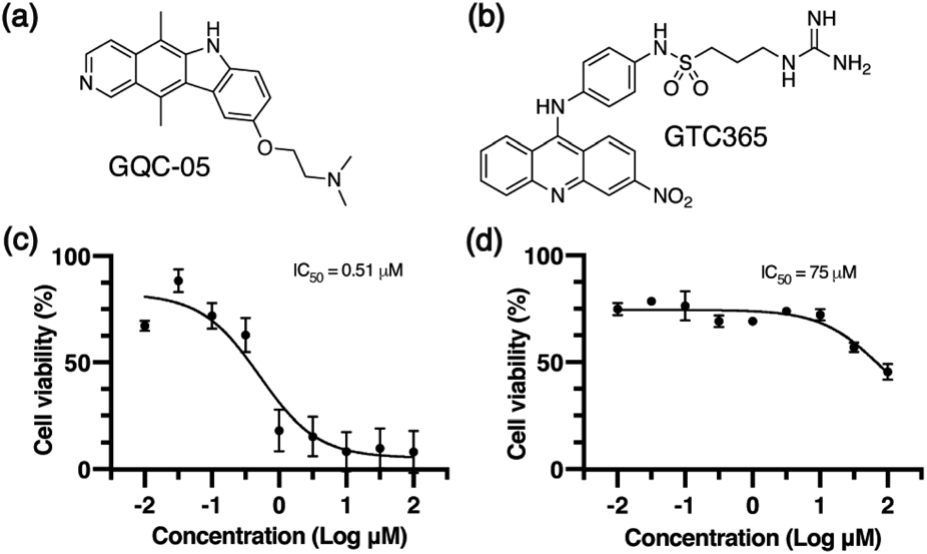
Small molecule G4-stabilising ligands. Molecular structures of (a) GQC-05 and (b) GTC365. Cell viability curves determined *via* MTS for MCF-7 cells after 72 h treatment with (c) GQC-05 and (d) GTC365.

We used two small molecule ligands, GQC-05 and GTC365, to stabilise G4s in MCF-7 human mammary epithelial adenocarcinoma cells and investigated the potential for G4 stabilisation to alter chromatin organisation and subsequent gene expression. GQC-05 (NSC338258, Fig. 1a) is an ellipticine analogue that binds with varying affinities to different G4-forming sequences, stabilising a range of G4 topologies.^33^ On the other hand, GTC365 (Fig. 1b) specifically binds to the higher-order G4 structure observed in the *hTERT* promoter *via* dual-motif targeting for the G4 and the mismatched duplex stem loop.^34^

We first evaluated the effects of 72 h incubation with each of the two G4 ligands on the viability of MCF-7 cells by an MTS assay (Fig. 1c and d). IC_50_ values for GQC-05 and GTC365 were found to be 0.51 μM and 75 μM, respectively, indicative of a higher toxicity associated with the broad targeting of GQC-05. It has previously been shown that treatment with G4-stabilising ligands can induce DNA damage,^35^ representing a mechanism whereby the broad-targeting G4 ligand, GQC-05, may cause greater G4 stabilisation than GTC365, resulting in increased DNA damage and higher toxicity. For further experiments, cells were incubated with the respective ligand at a concentration less than the IC_50_ for 72 h.

**Fig. 2:**
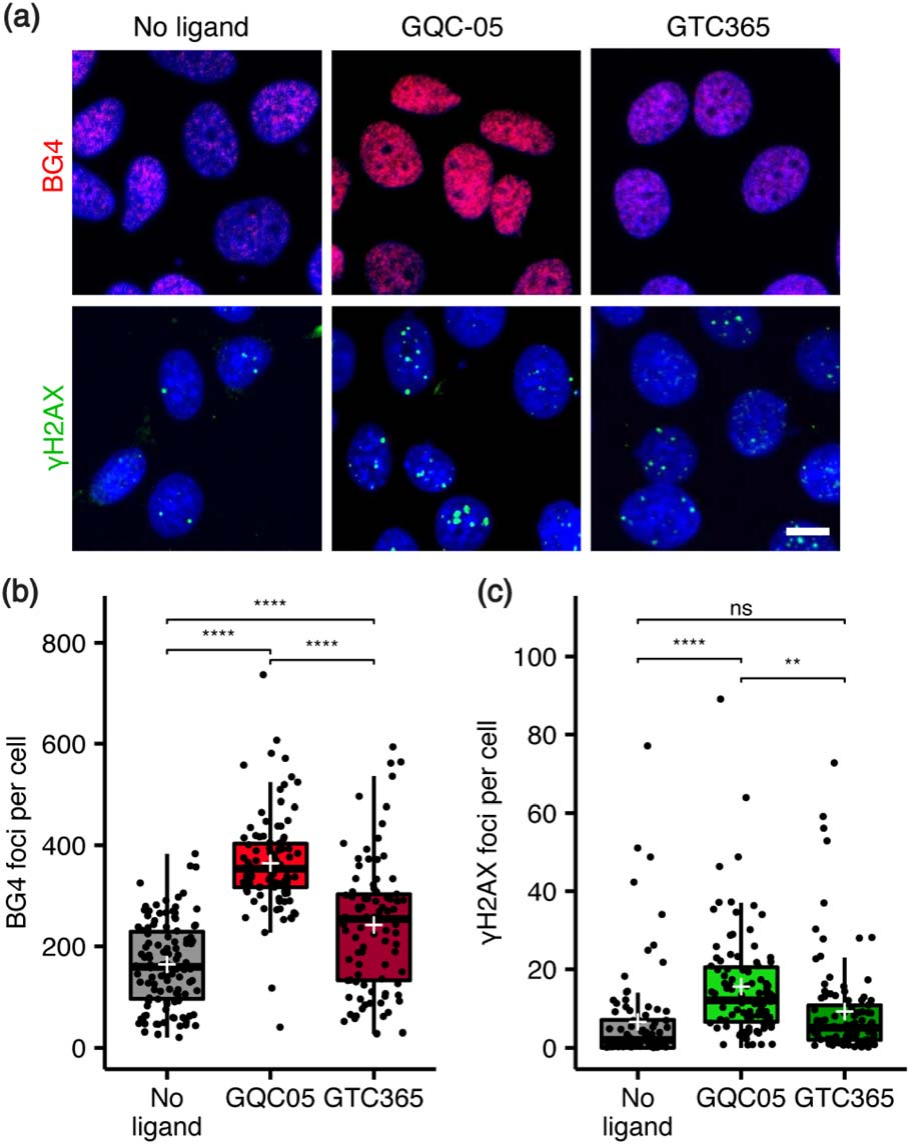
Effects of GQC-05 and GTC365 on nuclear G4 stabilisation and induction of DNA damage. (a) Representative images of immunocytochemistry of ligand-treated MCF-7 cells using BG4 (red) and γH2AX (green) antibodies, with nuclei counterstained with Hoechst 34580 (blue); Plan Apo 60×/1.40 objective; scale bar 10 μm. (b) The number of BG4 foci were counted and found to be greatest in GQC-05-treated cells, with GTC365 also increasing foci counts relative to the control (*n* > 90 for each sample). (c) GQC-05 also causes an increase in γH2AX foci, corresponding to instances of DNA damage, while GTC365 did not induce a significant change (*n* > 90 for each sample). **p < 0.01, ****p < 0.0001.

Next, the relationship between ligand targeting, G4 stabilisation and the induction of DNA damage was investigated *via* immunocytochemistry. G4s were identified with the structure-specific, single-chain variable fragment (scFv) BG4 antibody,^36^ and DNA damage identified using an antibody against γH2AX, a marker for DNA double-strand breaks.^37^ The broad-targeting G4 ligand, GQC-05, resulted in a significant increase in both γH2AX and BG4 foci counts, while the *hTERT* G4-targeting ligand, GTC365, resulted in a smaller increase in BG4 foci and no significant change in DNA damage (Fig. 2). Interestingly, GTC365 increased the mean BG4 foci count by 78 relative to the untreated control, suggesting it causes stabilisation of G4s other than the *hTERT* promoter G4 either directly or *via* downstream effects. The stabilisation of more G4s and greater induction of DNA damage by GQC-05 compared to GTC365 demonstrates that the induction of DNA double-stranded breaks is related to the extent of G4 stabilisation and thus the targeting specificity of the ligand, contributing to the higher toxicity of GQC-05. Our results mirror the findings of De Magis *et al.* where treatment with G4 stabilising ligands *in vivo* led to an increase in γH2AX foci, compared to non-G4 binders.^35^

**Fig. 3:**
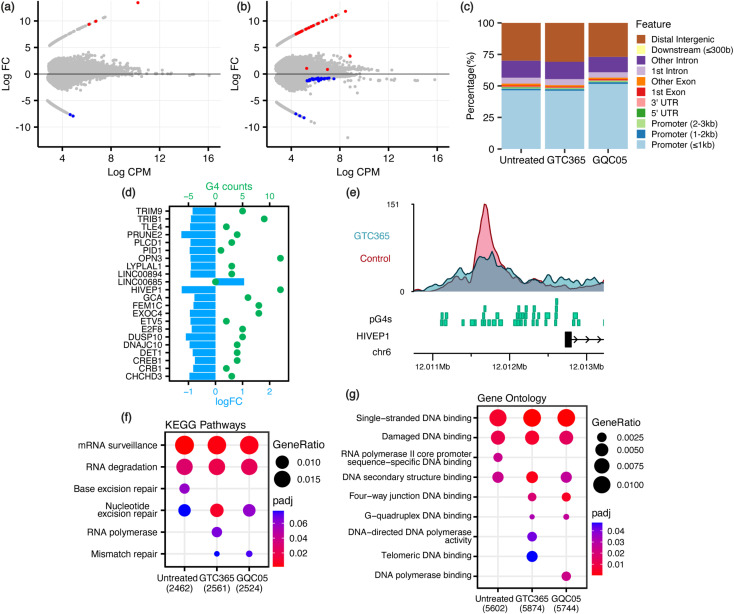
ATAC-seq analysis for GQC-05- and GTC365-treated MCF-7 cells. Mean-difference (MD) plots for (a) GQC-05 and (b) GTC365 treatments with differentially accessible regions shown in red (increased accessibility) and blue (decreased accessibility). FC, fold change; CPM, counts per million. (c) Feature distribution of ATAC-seq peaks in genomic regions shows increased promoter accessibility following GQC-05 treatment. (d) Predicted G4 (pG4) counts and change in chromatin accessibility (log_2_ FC) for the subset of 22 DARs located within gene promoter regions in GTC365-treated MCF-7 cells. Dots represent pG4 counts, and bars show the relative accessibility of the ATAC peak. All 21 regions with reduced accessibility contain pG4 sequences, while the single region with increased accessibility has no pG4s. (e) Distribution of ATAC peaks and pG4s in the promoter region of *HIVEP1*. The genomic positions of pG4 structures (green), the HIVEP1 gene (black), and ATAC peaks from the control (pink) and GTC365 (blue) ATAC-seq datasets are shown. Filtered DNA- and RNA-related (f) Kyoto Encyclopedia of Genes and Genomes (KEGG) pathway analysis and (g) Gene Ontology analysis of genes containing ATAC peaks in the promoter region, with colour corresponding to the adjusted *p*-value and dot size corresponding to the ratio of genes from the dataset associated with each pathway.

Given that remodelling of the chromatin landscape is a fundamental feature in the regulation of DNA damage response, we hypothesised that GQC-05 treatment would induce a significant shift in the global chromatin landscape, as opposed to GTC-365 which is expected to have more targeted effects. ATAC-seq analysis of ligand-treated MFC-7 cells showed differential chromatin accessibility in both GQC-05- and GTC365-treated samples. GQC-05 treatment resulted in a greater global shift of chromatin accessibility than GTC365, with accessibility of the promoters increasing to 51.7% of total peaks, 5% higher than the untreated control (46.6%) and GTC365 (46.2%) (Fig. 3 and S1, S2†). This is consistent with the well-characterised enrichment of G4-forming sites in gene promoter regions^10,38^ and parallels the observed extent of ligand-induced G4 stabilisation, as GQC-05 also caused a larger increase in BG4 foci counts than GTC365. Incubation with GTC365 induced 61 significant differentially accessible regions (DARs), defined as regions with false discovery rate (FDR) < 0.1 to allow for the largest range of genomic interactions to be identified. Of these 61 DARs, 22 were located in gene promoter regions. On the other hand, GQC-05 treatment resulted in only 5 DARs (Fig. 3a, b and Table S1†). This suggests that the stabilisation of specific G4s by GTC365 causes targeted alterations to chromatin accessibility that are consistent across the entire population of cells, resulting in significant changes for the target regions with minimal global implications. GQC-05, however, broadly targets a range of G4s, resulting in global shifts in accessibility towards gene promoters with few regions consistently affected across the entire cell population, resulting in fewer statistically significant DARs.

To explore the role of G4s in regulating chromatin accessibility changes, predicted G4 forming sequences (pG4s) within the genome were identified using the G4Hunter algorithm.^38^ The pG4 and ATAC-seq datasets were intersected to identify pG4s within promoter regions exhibiting differential accessibility (Fig. 3d, e and S3†). For GTC365, there is a strong correlation between pG4s and modified accessibility, with 21 (95.5%) of the 22 promoter DARs having both reduced accessibility and at least one pG4 (mean 5, range 1–12, median 4). For comparison, of all the ATAC-seq peaks detected in gene promoter regions, only 80.9% contained a pG4. This corresponds to *P* = 0.058 (binomial) for at least 21 out of 22 randomly selected regions to contain a pG4. The single DAR without a pG4 has increased accessibility. In contrast, 25 out of 39 non-promoter DARs had increased accessibility and 14 were reduced (Fig. S4†). Importantly, only 8 of the non-promoter DARs contained a pG4, and 7 of these 8 had reduced accessibility, consistent with the reduced accessibility observed for pG4-containing promoter DARs. Of note, GTC365 is reported to be specific for the *hTERT* promoter G4 *via* dual-motif targeting of the G4 and the mismatched duplex stem-loop,^34,39^ and thus the reduced accessibility of these pG4-containing promoter and non-promoter regions is likely associated with the G4-stabilising effects of GTC365. GQC-05 treatment resulted in only 5 DARs, with none located within gene promoters. The prevalence of pG4s in promoter regions with differential accessibility indicates that these pG4s may contribute to the modified chromatin landscape. Endogenous G4s are more commonly found in regions with high chromatin accessibility and are associated with highly transcribed genes,^18^ however ligand-induced stabilisation of G4s is predominately associated with transcriptional repression.^10,15,40,41^ Therefore, it is not surprising that ligand-induced G4 stabilisation with GTC365 results in a decrease in chromatin accessibility in gene promoters and in non-promoter regions containing a pG4.

**Fig. 4:**
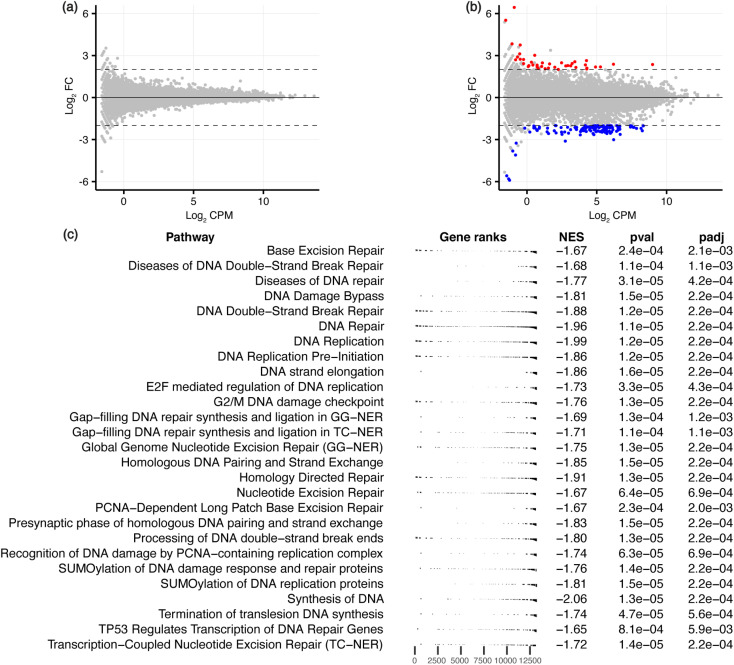
RNA-seq analysis for GQC-05- and GTC365-treated MCF-7 cells. MD plots for (a) GQC-05 and (b) GTC365 treatments with differentially expressed genes in red (upregulated) and blue (downregulated). (c) Gene set enrichment analysis (GSEA) of the GTC365 RNA-seq data, filtered to show pathways associated with DNA associated processes. The complete data is provided in Table S3 and Fig. S5.† The distribution of genes associated with each pathway is indicated (gene ranks), as is the normalised enrichment score (NES) and adjusted *p*-value.

To characterise the effects on the transcriptome and downstream biological processes, RNA-sequencing was conducted on ligand-treated MCF-7 cells, with significant differentially expressed genes (DEGs) defined using the threshold FDR < 0.05 and |log_2_ FC| > 2 to identify genes that are maximally altered by the ligands. GQC-05 did not cause any significant differential expression, similar to the low number of DARs induced by this broad-targeting G4 ligand. GTC365 treatment resulted in 162 DEGs (Fig. 4a, b, S1 and Table S2†), with 40 genes upregulated and 122 downregulated, consistent with the reported transcriptional suppression by ligand-induced stabilisation of G4s.^40,41^ This further supports the hypothesis that the higher specificity of GTC365 causes consistent stabilisation of the same G4s across the entire population of cells, resulting in a strong effect on accessibility of these sites and altered gene expression, while the broad-targeting G4 ligand, GQC-05, results in less consistent stabilisation across the population of cells. This is illustrated by the levels of *hTERT* expression, with GTC365 causing a 6.5-fold decrease in expression due to the specificity of the ligand for the *hTERT* promoter G4, while the broad-targeting G4 ligand, GQC-05, had negligible effects (Table S2†). Interestingly, despite the reported specificity of GTC365 for the *hTERT* G4, we observed differential expression of numerous other genes, that may be a result of off-target binding or downstream effects of ligand binding.

The lack of differential gene expression induced by GQC-05 was unexpected as it stabilises a wide range of G4s within cells and has been associated with differential gene expression over shorter time periods and at higher concentrations.^33,42^ However, for 72 h treatment as in this work, GQC-05 has relatively high toxicity and was therefore used at a low concentration to maintain cell viability. At this concentration, it is likely that the number of G4-ligand complexes formed is insufficient to stabilise all possible targets in each cell, with over 700 000 genomic G4-forming sequences having been experimentally validated.^9^ Additionally, Brown *et al.* demonstrated that for the *MYC* promoter G4, for which GQC-05 has the highest reported affinity, the GQC-05 : G4 stoichiometry is 2 : 1, indicative of two ligand binding sites per G4 for complete stabilisation.^33^ The binding constants for these two binding sites are 0.1 μM and 1.43 μM, so both the IC_50_ (0.51 μM) and the concentration used in this study (0.3 μM) will not result in consistent G4 stabilisation across cells. Consequently, different subsets of G4s may be stabilised in each cell, resulting in a reduced average effect on the genomic architecture and gene expression that may be better identified *via* single-cell studies rather than bulk RNA- or ATAC-seq. While higher doses of GQC-05 have been shown to induce transcriptomic modifications over short treatment periods,^33,42^ GQC-05 cytotoxicity varies significantly between cell types, and the lower dose treatments have different effects on gene expression in different cell lines.^33,43^

To explore the biological processes potentially affected by treatment with each ligand, pathway analysis was conducted on the ATAC- and RNA-seq results. Kyoto Encyclopedia of Genes and Genomes (KEGG) pathway analysis and Gene Ontology (GO) enrichment analysis was conducted on genes containing ATAC-seq peaks in the promoter region, and Gene Set Enrichment Analysis (GSEA) performed on all genes identified *via* RNA-seq, ranked by their expression levels. Many pathways were identified as differentially enriched by the two ligands, including several associated with DNA and RNA processes (Fig. 3f, g, 4c, S5 and Table S3†). Of note, GO analysis identified differential enrichment of GO terms associated with G-quadruplex binding and DNA polymerase activity in the ligand-treated samples, consistent with the stabilisation of G4s and their documented interactions with polymerases.^44–46^ Enrichment of these terms indicates that G4 stabilisation promotes a cellular response to regulate interactions with these structures. Furthermore, KEGG pathway analysis and GSEA demonstrate that both GTC365 and GQC-05 alter nucleotide excision- and DNA repair-related pathways, and GTC365 also caused differential enrichment of many pathways associated with RNA polymerase and DNA damage response, replication, and synthesis. As is expected for molecules that directly interact with DNA to modulate the chromatin landscape, there is a strong cellular response to both ligands, with many pathways involved in the maintenance of chromatin significantly affected. These results are in accordance with the immunocytochemistry experiments that demonstrated the ligands affect genomic stability and structure. GTC365 also alters several pathways related to cell cycle progression, which is halted in response to DNA damage and has also been associated with G4 stabilisation.^47^ It remains unclear whether these pathways are modified due to the ligands directly altering the accessibility and expression of the related genes, or if these pathways are stimulated as part of a biological response to the ligand treatment.

To explore if the transcriptomic effects of G4 stabilisation are due to altered accessibility of specific transcription factor (TF) binding sites, we conducted HOMER analysis on the ATAC-seq peaks to identify enriched TF binding motifs.^48^ Similar motifs were found to be enriched in each dataset, including the control (Tables S4 and S5†). Analysis of the 61 DARs from the GTC365-treated cells also identified these same motifs, suggesting that modified nucleosome occupancy of specific transcription factor binding motifs is not the primary mechanism by which these G4-stabilising ligands alter transcription. Instead, the transcriptional alterations may be a result of the interactions of stabilised G4s with DNA damage response pathways, or G4 structural effects. Additionally, the transcriptional effects of G4 ligands have previously been associated with altered helicase activity^49^ and their role as transcription factor binding sites,^17,18^ which can be impaired by G4 stabilisation.^41^

## Conclusions

In this work we have demonstrated that treatment of MCF-7 cells with G4-stabilising ligands alters the genomic landscape, transcriptome, and DNA damage response. Modifications in chromatin accessibility are observed to correspond to the extent of G4 stabilisation and targeting specificity of the G4 ligands. GTC365, which has been reported to target a specific G4, induced consistent, significant differential accessibility of certain regions of the genome, while the broad-targeting G4 ligand, GQC-05, caused general shifts in the chromatin landscape that increased accessibility of gene promoter regions. We identified novel GTC365-induced decreases in promoter accessibility that agrees with the documented transcriptional repression associated with ligand-induced G4-stabilisation. Unlike GQC-05, GTC365 caused significant differential gene expression with the majority of altered genes being downregulated. This represents the dual possibility of small molecule G4-stabilising ligands as therapeutic agents to target G4s in diseases, inducing either specific or global effects depending on the design of the molecule.

## Data availability

The raw data reported in this paper are available at the NCBI GEO repository. Processed data files are included in the ESI.†

## Author contributions

A. O. and J. J. K. performed cell culture experiments and A. O. prepared samples for sequencing. N. B. L. conducted sequencing analysis and bioinformatic predictions. N. M. S., K. S. I. and C. W. E. developed the concept. All authors discussed and analysed results and contributed to the manuscript.

## Conflicts of interest

There are no conflicts to declare.

## Supplementary Material

SC-014-D3SC02533K-s001

SC-014-D3SC02533K-s002

SC-014-D3SC02533K-s003

SC-014-D3SC02533K-s004

SC-014-D3SC02533K-s005
