# Supplementary material for: G4-DNA formation and chromatin remodelling are interdependent in human cells
Source: Chem Sci. 2023 Jun 27;14(28):7681–7. doi: 10.1039/d3sc02533k (PMC10355101; doi:10.1039/d3sc02533k)
Supplement: SC-014-D3SC02533K-s001 [file SC-014-D3SC02533K-s001.pdf]

Electronic Supplementary Material (ESI) for Chemical Science.
This journal is © The Royal Society of Chemistry 2023

Supporting Information

G4-DNA Formation and Chromatin Remodelling are Interdependent in Human Cells

Nicholas B. Lawler[1,2,‡], Arnold Ou[1,‡], Jessica J. King[1], Cameron W. Evans[1,*], K. Swaminathan Iyer[1,*], and Nicole M. Smith[1,*]

[1] School of Molecular Sciences, The University of Western Australia, Perth, WA, Australia.
[2] School of Physics, Mathematics and Computing, The University of Western Australia, Perth, WA, Australia.
‡ These authors contributed equally.
*cameron.evans@uwa.edu.au
*nicole.smith@uwa.edu.au
*swaminatha.iyer@uwa.edu.au

Methods

G4 stabilising compounds were provided by L. Hurley (Arizona, USA).

Cell culture

MCF-7 human epithelial breast adenocarcinoma cells were cultured in Minimum Essential Medium α (MEMα, Gibco, 12571063) supplemented with 10% v/v foetal bovine serum (FBS, Gibco, 10437028), 1% v/v sodium bicarbonate 7.5% w/v solution (Gibco, 52080094), and 1x GlutaMAX (Gibco, 35050061). No antibiotic/antimycotic agents were used. Cells were maintained in a humidified incubator at 37 °C, 5% CO_2, and passaged at 80% confluency with 1x TrypLE Select (Gibco, 12563011) for sequencing or 1x TrypLE Express (Gibco, 12604039) for staining.

IC_{50} determination

MCF-7 cells were seeded at a density of 2,000 cells per well in 96-well plates the day prior to experiments and incubated overnight. Ligands were diluted in complete culture medium to final concentrations in the range 0.01–100 µM. 100 µl of these solutions were applied to the cells for 72 h, then 40 µl MTS (CellTiter 96 AQueous Cell Proliferation Assay, Promega) was added for 2 h. Optical density was recorded at 490 nm using a 2300 EnSpire Multimode Plate Reader. The data were analysed using GraphPad Prism v8.4.

BG4 immunofluorescence

G4 structures in the nuclei were targeted with the structure-specific FLAG-tagged scFv-BG4 antibody (MABE917, Millipore). MCF-7 cells were seeded at 100,000 cells/well on 12 mm^2 sterile coverslips in a 12-well plate and incubated overnight at 37 °C and 5% CO_2 in complete media. Control samples were incubated in complete media under the same conditions. Samples were treated with G4 ligands at approximately the IC_{40}; 0.3 µM GQC-05, 20 µM GTC365 for 72 h at 37 °C and 5% CO_2. Cells were prefixed with 4% w/v paraformaldehyde (PFA) for 2 min at room temperature. The media was removed, cells were washed once with 0.1% v/v Triton X-100/PBS (Sigma-Aldrich, T8787) and then with PBS. Cells were incubated with cytoplasm removal buffer (20 mM HEPES-KOH pH 7.9, 20 mM NaCl, 5 mM MgCl2, 300 mM sucrose, 0.5% v/v NP-40 Surfact-Amps® Detergent Solution (Thermo Fisher, 85124)) for 15 min at room temperature. Cells were fixed with 4% w/v PFA for 20 min at room temperature, washed with PBS, and then permeabilised with 0.1% v/v Triton X-100/PBS for 10 min. Samples were blocked with 2% w/v skim milk in PBS for 1 h at room temperature to prevent non-specific binding then washed thrice with PBS. Samples were incubated with FLAG-tagged BG4 antibody (300 nM in 1% w/v milk/PBS) for 1 h at 37 °C. Cells were washed with 0.1% v/v Tween 20 (Sigma-Aldrich, P9416) in PBS followed by incubation with rabbit anti-Flag antibody (1/1000 in 1% w/v skim milk in PBS) (DYKDDDDK tag antibody, Cell Signaling Technologies, 2368) for 1 h at 37 °C. Coverslips were washed three times with 0.1% v/v Tween 20/PBS, followed by incubation with donkey anti-rabbit Alexa Fluor 594 (1/2000, Invitrogen, A21207) and Hoechst 34580 (1 µg/mL, Sigma-Aldrich, 63493) in 1% milk/PBS at 37 °C for 30 min. Coverslips were washed once with 0.1% v/v Tween20/PBS and PBS then mounted on clean microscope slides using Fluoromount G (Invitrogen, 00-4958-02). Images were acquired with a Nikon A1 RMP confocal microscope (Plan Apo VC 60x/1.4) using laser wavelengths 405 nm and 561 nm. Images were analysed using ImageJ. Statistical comparisons were performed performed using one-way ANOVA and multiple comparisons tests with GraphPad Prism.

γH2AX Immunofluorescence

MCF-7 cells were seeded at 100,000 cells/well on 12 mm^2 sterile coverslips in a 12-well plate and incubated overnight at 37 °C and 5% CO_2 in complete media. Control samples were incubated in complete media under the same conditions. Samples were treated with G4 ligands at approximately the IC_{40}; 0.3 µM GQC-05, 20 µM GTC365 for 72 h at 37 °C and 5% CO_2. Media was removed gently, and cells were washed with PBS once. Cells were fixed with 1:1 methanol: acetone for 20 min at -20 °C. Next, cells were washed with PBS once and permeabilised with 0.1% v/v TritonX/PBS for 15 min at room temperature, followed by two 5 min PBS washes. Cells were blocked with 3% BSA/PBS for 1 h, then incubated with rabbit anti-γH2AX antibody (Abcam, ab11174) diluted 1/500 in 1% BSA/PBS for 2 h at room temperature. Coverslips were washed 3 times with 0.1% v/v Tween 20 in PBS and incubated with goat anti-rabbit Alexa Fluor 488-conjugated IgG antibody (1/1000 in 1% BSA/PBS) for 1 h at room temperature, followed by two washes with 0.1% v/v Tween 20/PBS. Cells were incubated with Hoechst (1 µg/mL) diluted in PBS for 15 min at room temperature and washed twice with PBS before mounting onto microscope slides with Fluoromount G. Images were acquired with a Nikon A1 RMP confocal microscope (Plan Apo VC 60x/1.4), using laser wavelengths 405 nm and 488 nm. Images were analysed using ImageJ. Statistical comparisons were performed using one-way ANOVA and multiple comparisons tests with GraphPad Prism.

Ligand incubation

G4 ligand solutions of 100 mM in DMSO were diluted to approximately the IC_{40} in a total of 10 ml MCF-7 complete media before each experiment (GQC-05 0.3 µM, GTC-365 20 µM). MCF-7 cells were seeded into 150 mm plates and allowed to adhere overnight. The following day, complete media was removed and replaced with 10 ml drug dilution in media. Controls received 10 ml fresh complete media. Cells were incubated for 72 h in the presence of ligands

before the medium was removed, monolayers washed with PBS, and cells harvested by detachment using TrypLE Select. Cells were collected by centrifugation for 5 min at 200*g* and counted using a haemocytometer.

ATAC-seq

ATAC-seq was conducted as previously described by Buenrostro *et al.*[1] Raw reads were processed by FastQC for quality control and adapters removed via Cutadapt. The reads were then aligned to the Genome Reference Consortium GRCh37.p13 (hg19) reference genome using BWA (Burrows-Wheeler Alignment, version 0.7.5)[2] and sorted using samtools. Duplicate reads were removed using Picard tools (http://broadinstitute.github.io/picard/). Peak calling was conducted on each individual sample using MACS2.[3] Peaks were intersected via bedtools[4] for downstream analysis. Differential peak analysis was conducted in R using the edgeR package,[5] with peak annotations via ChIPseeker[6] and pathway analysis via clusterProfiler.[7]

RNA-seq

Total RNA was extracted using RNeasy Mini Kit (Qiagen, 74106). RNA-seq libraries were then prepared using the TruSeq stranded total RNA with Ribo-Zero Human/Mouse/Rat library prep kit (Illumina) and sequenced using 50-bp single reads on the HiSeq2500 platform. Quality control of the fastq files was conducted using FastQC. The reads were mapped to the human reference genome (Genome Reference Consortium GRCh38/hg38) via STAR,[8] with annotations from GENECODE version 25. Differential expression analysis of the normalised reads was conducted in R using the edgeR package,[5] with gene set enrichment analysis conducted on the genes ranked by $\log_2 FC \times -\log_{10}(FDR)$ using the fgsea package.[9]

G4 prediction algorithm

G4Hunter[10] was run on the GRCh37 reference genome (hg19) using a sliding window of 25 base pairs and a score threshold of 1.6 to identify regions of DNA with the potential to form G4s in physiologically relevant conditions.

Motif analysis

Enriched motifs were identified from ATAC-seq peak files using findMotifsGenome.pl with the -size 200 option from HOMER.[11] Analysis was conducted on both the raw ATAC-seq peaks and the differentially accessible regions

Supplementary Figures

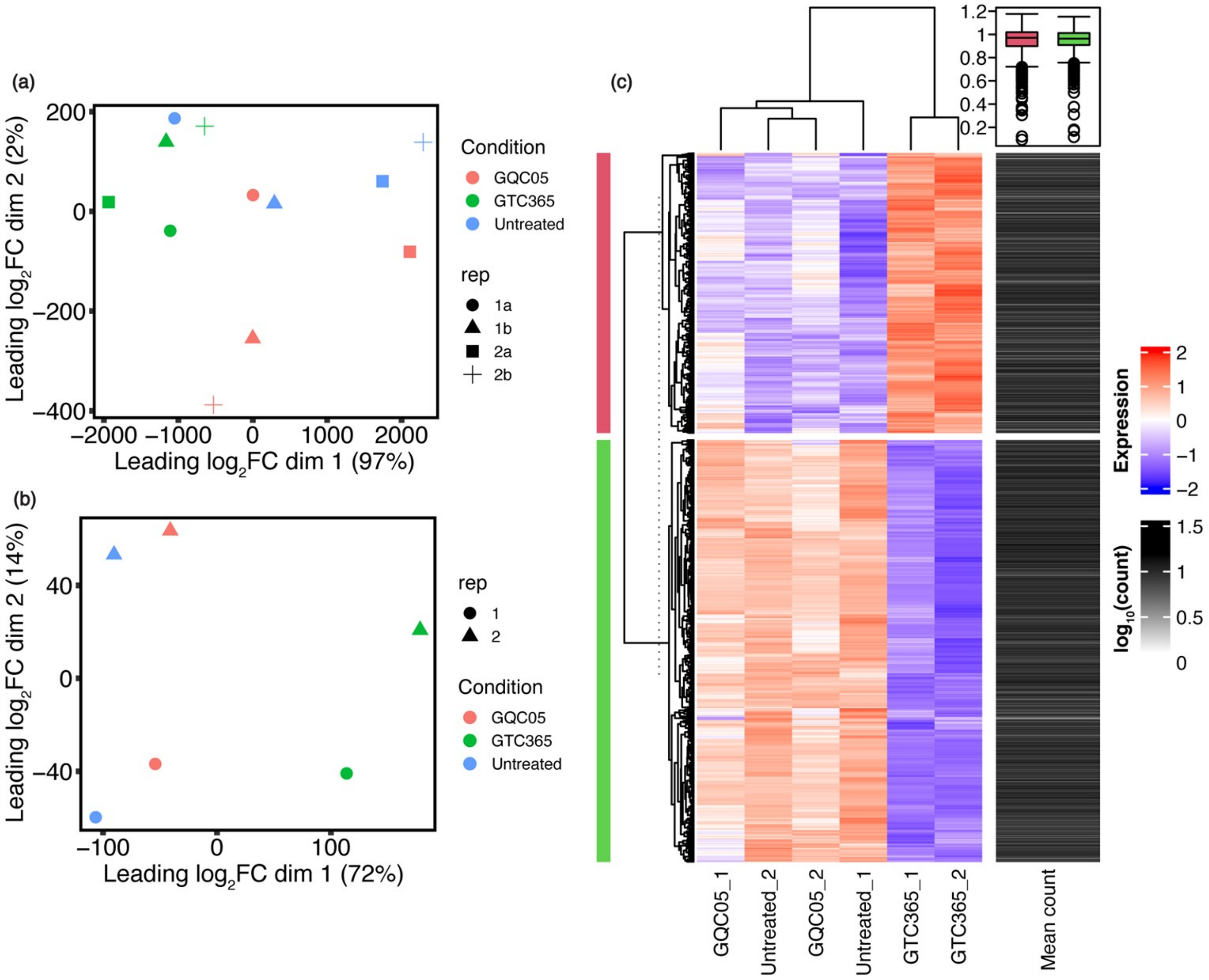

Fig. S1. Principal component analysis (PCA) of **(a)** ATAC-seq and **(b)** RNA-seq data from untreated, GQC-05 and GTC365-treated MCF7 cells. Dots represent samples, with condition indicated by colour and biological replicate by shape. The variance associated with each dimension is noted in brackets in the axis labels. **(c)** Heatmap showing relative expression and abundance of the top 2000 differentially expressed genes. Colour corresponds to the relative expression levels, while the intensity of the mean count column indicates the average abundance of the gene. The mean counts of up- and down-regulated genes are shown by pink and green groupings in the boxplot.

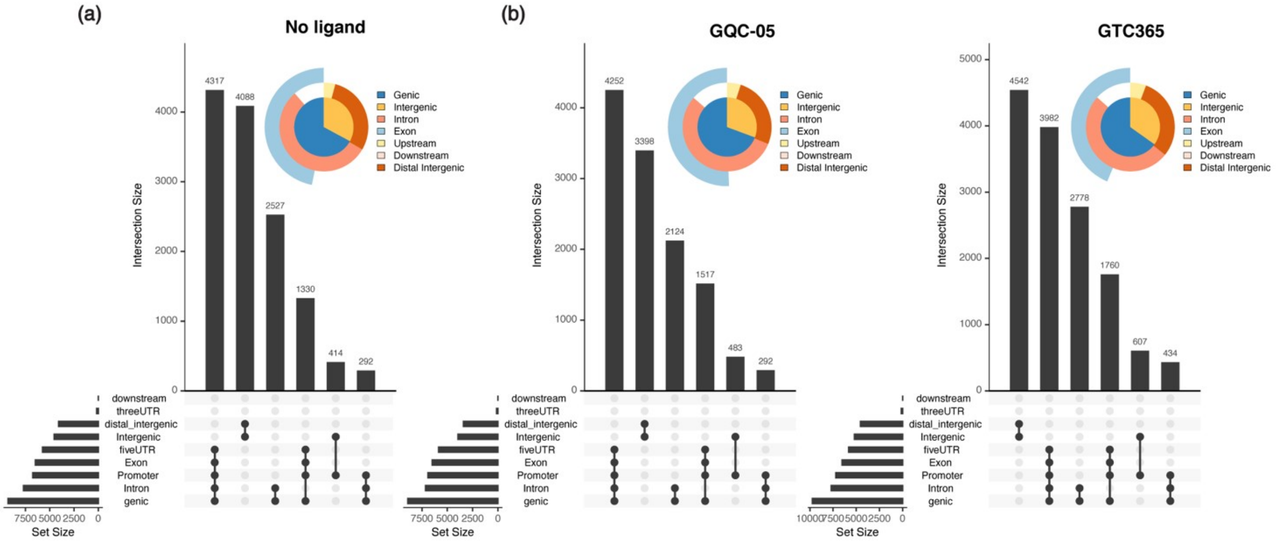

Fig. S2. Upset plots showing the distribution of ATAC-seq peaks in genomic regions for **(a)** control, **(b)** GQC-05 and **(c)** GTC365 treated MCF7 cells.

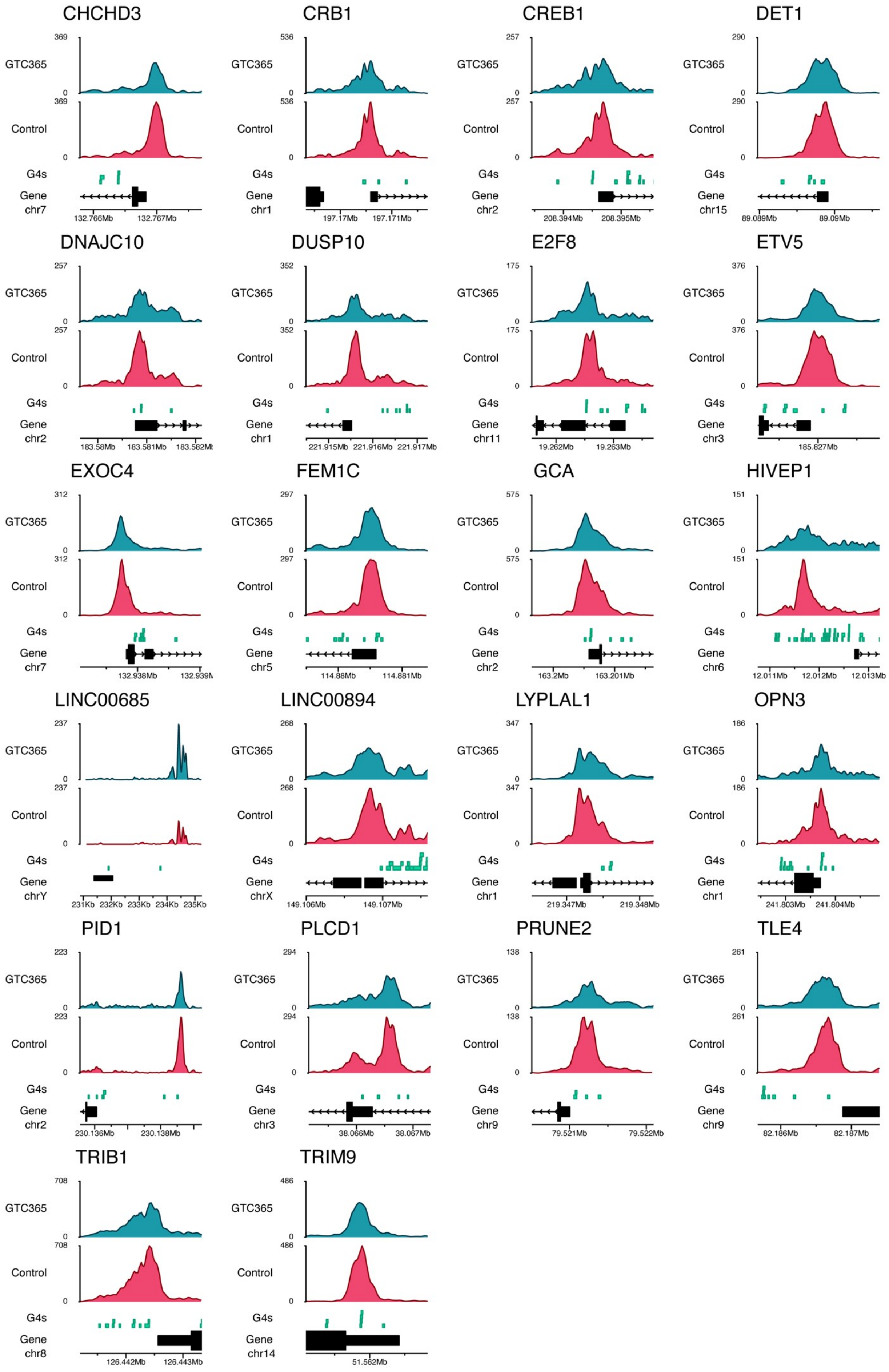

CHCHD3
CRB1
CREB1
DET1
DNAJC10
DUSP10
E2F8
ETV5
EXOC4
FEM1C
GCA
HIVEP1
LINC00685
LINC00894
LYPLAL1
OPN3
PID1
PLCD1
PRUNE2
TLE4
TRIB1
TRIM9
GTC365
Control
G4s
Gene
chr7
chr1
chr2
chr15
chr11
chr3
chr5
chr6
chrY
chrX
chr9
chr8
chr14
132.766Mb
132.767Mb
197.17Mb
197.171Mb
208.394Mb
208.395Mb
89.089Mb
89.09Mb
183.58Mb
183.581Mb
221.915Mb
221.916Mb
221.917Mb
19.262Mb
19.263Mb
185.827Mb
132.938Mb
114.88Mb
114.881Mb
163.2Mb
163.201Mb
12.011Mb
12.012Mb
12.013Mb
231Kb
232Kb
233Kb
234Kb
235Kb
149.106Mb
149.107Mb
219.347Mb
219.348Mb
241.803Mb
241.804Mb
230.136Mb
230.138Mb
38.066Mb
38.067Mb
79.521Mb
79.522Mb
82.186Mb
82.187Mb
126.442Mb
126.443Mb
51.562Mb

Fig. S3. Distribution of ATAC peaks and G4s of the 22 promoter DARs for GTC365. Peaks from the control (pink) and GTC365 (blue) ATAC-seq datasets are shown. The genomic positions of predicted G4 structures (pG4s, green), the associated gene (black) and ATAC peaks from the control (pink) and GTC365 (blue) ATAC-seq datasets are shown.

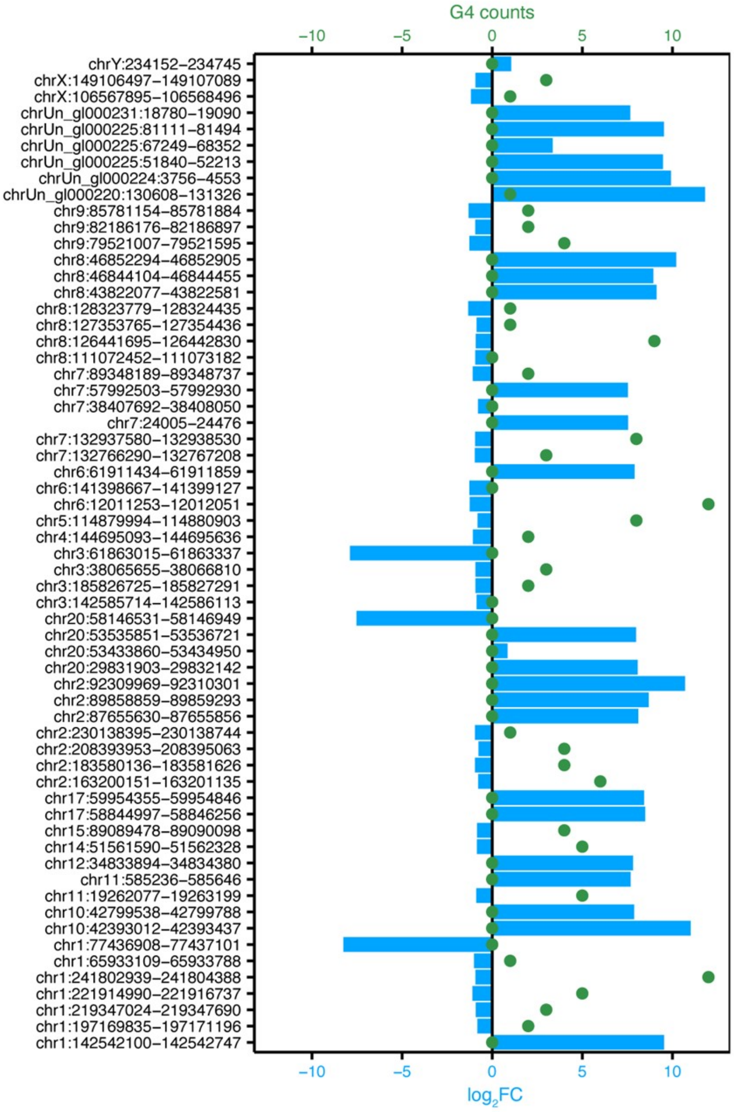

Fig. S4. G4 counts and change in chromatin accessibility (log_2FC) for all 61 DARs in GTC365-treated MCF-7s. Dots represent G4 counts, and bars show the relative accessibility of the ATAC peak.

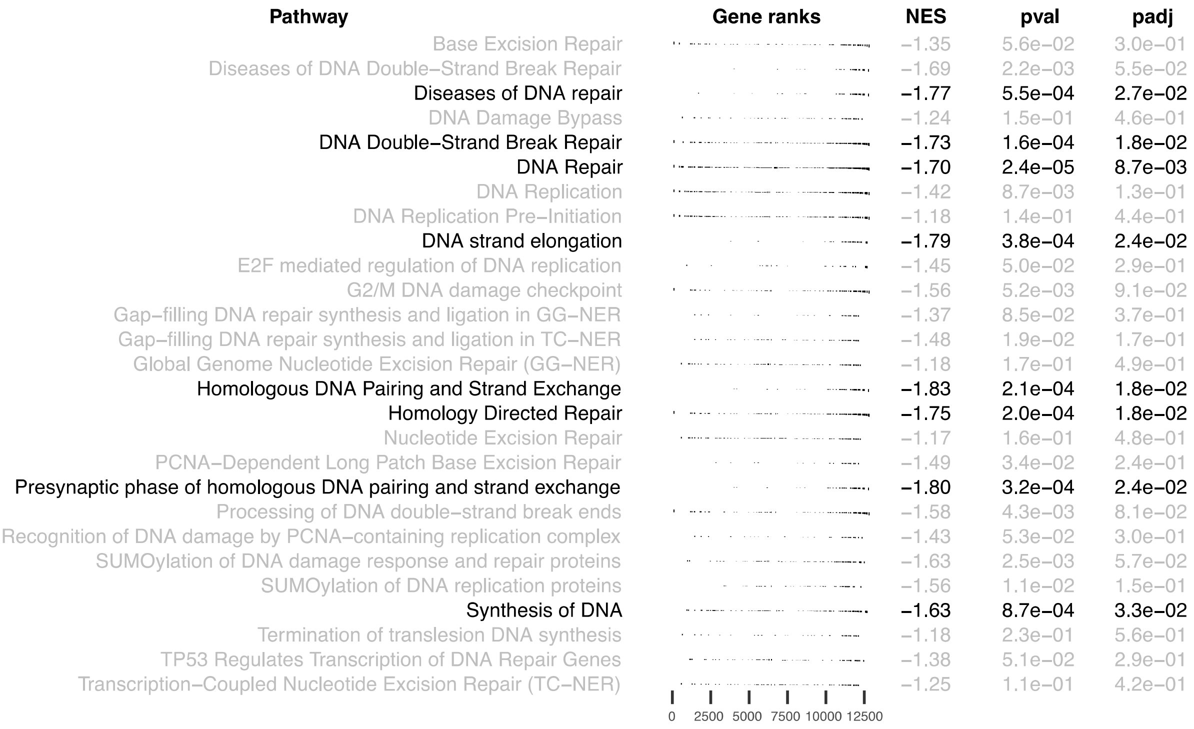

Fig. S5. Gene set enrichment analysis (GSEA) of GQC-05 RNA-seq data, filtered to show pathways associated with DNA associated processes. The complete data is represented in Table S3. The distribution of genes associated with each pathway is indicated (gene ranks), as is the normalized enrichment score (NES) and adjusted *p*-value.

Supplementary Tables

Table S1. Differentially accessible regions (DARs) from ATAC-seq analysis. Provided as separate .xlsx file.

Table S2. Differentially expressed genes (DEGs) from RNA-seq analysis. Provided as separate .xlsx file.

Table S3. Gene set enrichment analysis table. Provided as separate .xlsx file.

Table S4. Motif analysis data. Provided as separate .xlsx file.

Table S5. Top 15 motifs identified from the ATAC-seq peak files using HOMER. The number of target sequences containing each motif and the percentage of total sequences is reported for each of the three conditions.

		Target sequences with motif			Percentage sequences with motif		
Motif	Name	Untreated	GQC-05	GTC365	Untreated	GQC-05	GTC365
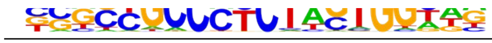	CTCF(Zf)/CD4+-CTCF-ChIP-Seq(Barski_et_al.)/Homer	13572	11619	11329	13.32%	12.66%	10.57%
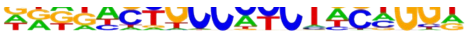	BORIS(Zf)/K562-CTCFL-ChIP-Seq(GSE32465)/Homer	14883	13222	12837	14.61%	14.41%	11.98%
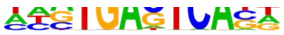	Fra2(bZIP)/Striatum-Fra2-ChIP-Seq(GSE43429)/Homer	9406	8548	9806	9.23%	9.32%	9.15%
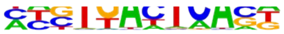	Fra1(bZIP)/BT549-Fra1-ChIP-Seq(GSE46166)/Homer	10303	9271	10690	10.11%	10.10%	9.98%
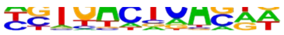	Fosl2(bZIP)/3T3L1-Fosl2-ChIP-Seq(GSE56872)/Homer	7641	7083	7998	7.50%	7.72%	7.46%
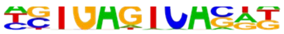	Jun-AP1(bZIP)/K562-cJun-ChIP-Seq(GSE31477)/Homer	6358	5940	6672	6.24%	6.47%	6.23%
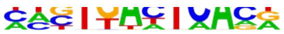	Fos(bZIP)/TSC-Fos-ChIP-Seq(GSE110950)/Homer	10610	9546	11013	10.41%	10.40%	10.28%
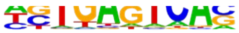	JunB(bZIP)/DendriticCells-Junb-ChIP-Seq(GSE36099)/Homer	10176	9168	10588	9.99%	9.99%	9.88%
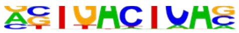	BATF(bZIP)/Th17-BATF-ChIP-Seq(GSE39756)/Homer	11005	9863	11476	10.80%	10.75%	10.71%
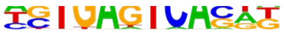	Atf3(bZIP)/GBM-ATF3-ChIP-Seq(GSE33912)/Homer	11100	10003	11562	10.90%	10.90%	10.79%
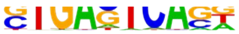	AP-1(bZIP)/ThioMac-PU.1-ChIP-Seq(GSE21512)/Homer	11649	10403	12126	11.43%	11.34%	11.32%
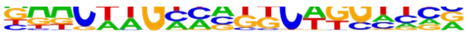	GRHL2(CP2)/HBE-GRHL2-ChIP-Seq(GSE46194)/Homer	6190	5176	5935	6.08%	5.64%	5.54%
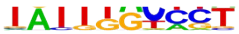	FOXM1(Forkhead)/MCF7-FOXM1-ChIP-Seq(GSE72977)/Homer	11903	10112	12658	11.68%	11.02%	11.81%
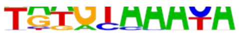	FOXA1(Forkhead)/MCF7-FOXA1-ChIP-Seq(GSE26831)/Homer	11506	9723	12462	11.29%	10.60%	11.63%
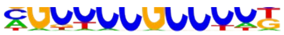	Sp1(Zf)/Promoter/Homer	11542	12507	10913	11.33%	13.63%	10.19%

References

1 J. D. Buenrostro, B. Wu, H. Y. Chang and W. J. Greenleaf, *Curr. Protoc. Mol. Biol.*, 2015, **109**, 21.29.1-21.29.9.
2 H. Li and R. Durbin, *Bioinformatics*, 2009, **25**, 1754–1760.
3 Y. Zhang, T. Liu, C. A. Meyer, J. Eeckhoute, D. S. Johnson, B. E. Bernstein, C. Nusbaum, R. M. Myers, M. Brown, W. Li and X. S. Liu, *Genome Biol.*, 2008, **9**, R137.
4 A. R. Quinlan and I. M. Hall, *Bioinformatics*, 2010, **26**, 841–842.
5 M. D. Robinson, D. J. McCarthy and G. K. Smyth, *Bioinformatics*, 2010, **26**, 139–140.
6 G. Yu, L.-G. Wang and Q.-Y. He, *Bioinformatics*, 2015, **31**, 2382–2383.
7 G. Yu, L.-G. Wang, Y. Han and Q.-Y. He, *OMICS J. Integr. Biol.*, 2012, **16**, 284–287.
8 A. Dobin, C. A. Davis, F. Schlesinger, J. Drenkow, C. Zaleski, S. Jha, P. Batut, M. Chaisson and T. R. Gingeras, *Bioinformatics*, 2013, **29**, 15–21.
9 G. Korotkevich, V. Sukhov and A. Sergushichev, *bioRxiv*, 2019, 060012.
10 A. Bedrat, L. Lacroix and J.-L. Mergny, *Nucleic Acids Res.*, 2016, **44**, 1746–1759.
11 S. Heinz, C. Benner, N. Spann, E. Bertolino, Y. C. Lin, P. Laslo, J. X. Cheng, C. Murre, H. Singh and C. K. Glass, *Mol. Cell*, 2010, **38**, 576–589.
